# Superior hypogastric nerve block (SHNB) for pain control after uterine fibroid embolization (UFE): technique and troubleshooting

**DOI:** 10.1186/s42155-020-00141-2

**Published:** 2020-09-27

**Authors:** Keith Pereira, Louis Maurice Morel-Ovalle, Mehdi Taghipour, Afsheen Sherwani, Roshni Parikh, Jerome Kao, Kirubahara Vaheesan

**Affiliations:** 1 Saint Louis University, 3635 Vista avenue, St. Louis, MO 63110 USA; 2 Einstein Medical Center Montgomery, East Norriton, PA USA

**Keywords:** Uterine fibroid embolization (UFE), Pain, Superior hypogastric nerve block (SHNB)

## Abstract

**Background:**

Superior Hypogastric nerve Block (SHNB) has been shown to be an effective pain management technique after Uterine Fibroid Embolization (UFE), reducing the need for opiates and allowing same-day discharge after UFE. In this technical note we discuss relevant anatomy and technical details in performing SHNB.

**Main body:**

The Superior hypogastric plexus (SHP) is the part of the abdominopelvic sympathetic nervous system that provides a targeted intervention to sympathetic-mediated pain pathways of pelvic organs and a target for an anterior approach Superior Hypogastric nerve Block after embolization. Vascular structures are in close relation to the intended site of target of the SHP at the L5 vertebral body include aortic bifurcation and IVC confluence, hence a detailed knowledge of this is essential. A step by step technical approach to SHNB includes patient positioning for the block, image guidance and needle positioning, choice and technique of anesthetic injection. Traversing a large fibroid uterus, inadvertent vascular opacification and Local anesthetic systemic toxicity present challenges to performing the block and are addressed.

**Conclusion:**

Superior Hypogastric nerve Block (SHNB) can be a useful tool in the Interventional armamentarium to make UFE a better experience for patients with fibroids, allowing for better pain control as well as facilitating same day discharge. Performing SHNB appear to be can be performed with technical ease for an interventional radiologist.

## Background

Superior Hypogastric nerve Block (SHNB) has been shown to be effective pain suppressant after UFE, reducing the need for opiates and allowing same-day discharge after UFE (Pereira et al. n.d.; Rasuli et al. 2004).

Performing intraprocedural SHNB appears to be technically straightforward for a trained Interventional radiologist (Pereira et al. n.d.). In this technical note we discuss relevant anatomy and technical details in performing SHNB. We also discuss the challenges associated with SHNB and suggest troubleshooting ideas, based on our experience.

### Clinically relevant anatomy

#### The superior hypogastric plexus (SHP) and anatomy

**Fig. 1:**
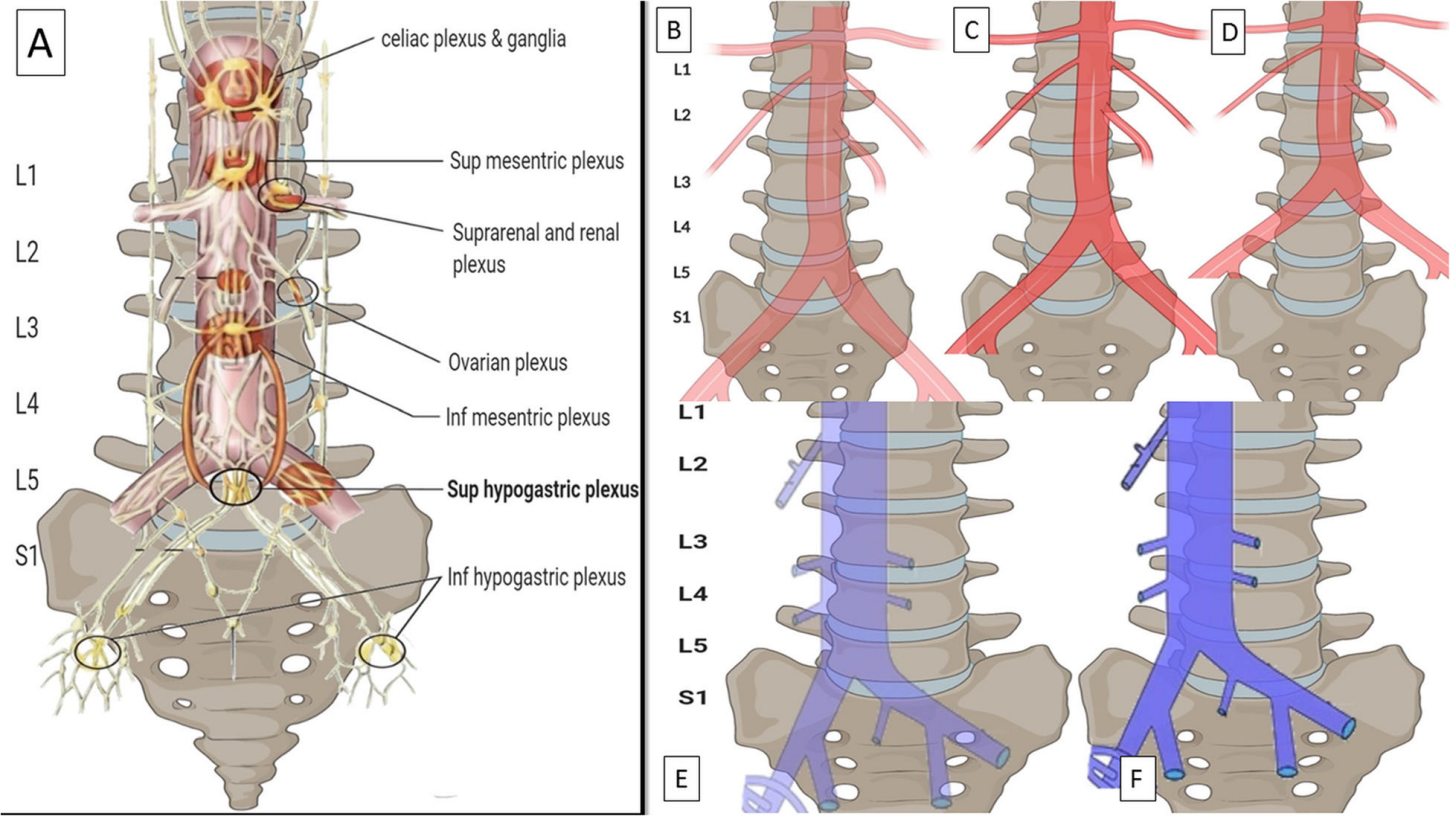
Demonstration of the SHP and the relevant radiographic and vascular anatomy as a guide to appropriate needle positioning and nerve blockade targeting during a SHNB. **a** The SHP (darker shade oval) targeted during the SHNB mainly lies at the L5 vertebra. **b, c, d** Aortic bifurcation and relevant radiographic anatomy. In majority of cases (63–80%) the bifurcation is at the level of L4 vertebra or L4–5 disc (**c**). B and D represent the less commonly seen high and low bifurcations. **e**, **f** Demonstration of iliocaval confluence and relevant radiographic anatomy. In 60–70% cases the iliocaval junction is seen between L4 and L5-S1 disc (E), specifically at the upper third and at right lateral third of the L5 vertebral body (F)

The Superior hypogastric plexus (SHP) is the part of the abdominopelvic sympathetic nervous system that allows a targeted intervention to sympathetic-mediated pain pathways of pelvic organs. SHP is a bilateral continuation of the paravertebral sympathetic chain and aortic plexus nerve fibers, forming a complex network of fibers surrounding the anterior and lateral aspects of the lower abdominal aorta (RJP and Wu 2014). (Fig. 1a).

Radiographically the SHP is a retroperitoneal and midline structure and is usually located anterior to the lower third of L5 and upper third of S1 (RJP and Wu 2014) is accessible for neural blockade in this area (Fig. 1a). As UFE is performed with the patient supine in the fluoroscopy room, an anterior, fluoroscopy guided approach appears optimal. This reduces chances of discitis from a posterior approach, however risk of vascular injury and intravascular injection remain.

#### Vascular structures

Vascular structures in close relation to the intended site of target of the SHP at the L5 vertebral body include aortic bifurcation and Inferior Vena Cava (IVC) confluence. Vascular anatomy can be evaluated on magnetic resonance images, if available. When performing UFE using a femoral approach it is easy to outline the aortic bifurcation after fluoroscopic evaluation of the catheter as it crosses over the aortic bifurcation. Radial artery access provides a unique challenge, which warrants the need for an aortogram to identify the aortic bifurcation. Several studies have shown that the aortic bifurcation lies between L3 and L5 vertebral levels (Fig. 1 B,C,D), the majority being at the level of L4 vertebra or L4–5 disc (63–80%) (Chithriki et al. 2002). The iliocaval junction however continues to be a challenge as it cannot be outlined with an angiogram and the IVC is also more prone to variation when compared to aorta. The iliocaval junction is seen between L4 and L5-S1 disc (Fig. 1 E,F), specifically on the body of L5 in about 60–70% cases. In relation to the aortic bifurcation, the iliocaval junction is in most cases usually at about 19 mm below it (Appaji et al. 2014).

### SHNB technique during UFE

#### Preparation and needle positioning

**Fig. 2:**
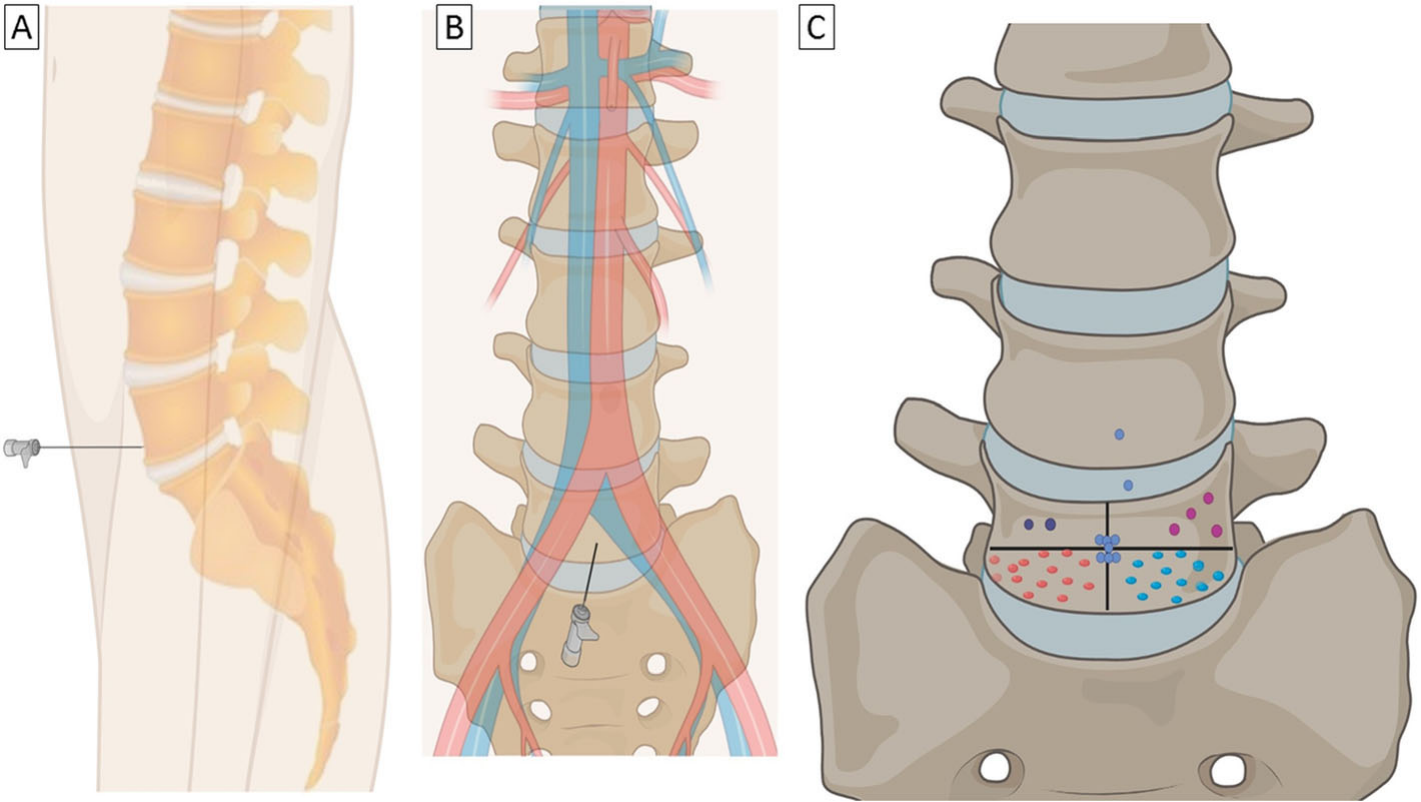
Demonstration of needle approach to the L5 vertebral body in Lateral (**a**) and AP (**b**) views. The relevant most common vascular anatomy has also been included in B. **c** The most commonly targeted sites in our single center experience. We typically target the inferior aspect of the L5 vertebral body in an attempt to stay distal to the aortic bifurcation. In review of target in our cases we actually targeted the left lower quadrant of the L5 body in 29% (12/41), right lower quadrant of the L5 body in 34% (14/41), midpoint l5 body in 17% (7/41), right upper quadrant in 4.8% (2/41), left upper quadrant in 9.7%(4/41) and lower L4 body in 4.8% (2/41)

Before starting UFE the entire lower abdomen is prepared in a surgical sterile fashion, in preparation of SHNB. UFE is then performed based on standard protocols. An abdominal aortogram is performed with the imaging intensifier panel in a cranio-caudal tilt (between 5 and 20 degrees) to identify the 5th vertebral body in a true antero-posterior view and outline the aortic bifurcation. The aortogram maybe skipped if prior imaging is available or in case of femoral access where the catheter over the bifurcation can help delineate the aortic bifurcation. We typically target the inferior aspect of the L5 vertebral body in an attempt to stay distal to the aortic bifurcation (Fig. 2). The targeted sites in descending order of frequency included right lower quadrant of the L5 body in 34% (14/41), left lower quadrant of the L5 body in 29% (12/41), midpoint l5 body in 17% (7/41), left upper quadrant in 9.7% (4/41), right upper quadrant in 4.8% (2/41) and lower L4 body in 4.8% (2/41). Based on this experience and the fact that the left common iliac vein was most often opacified (elaborated later in this paper), we now tend to favor targeting the right lower quadrant of L5 vertebral body.

**Fig. 3:**
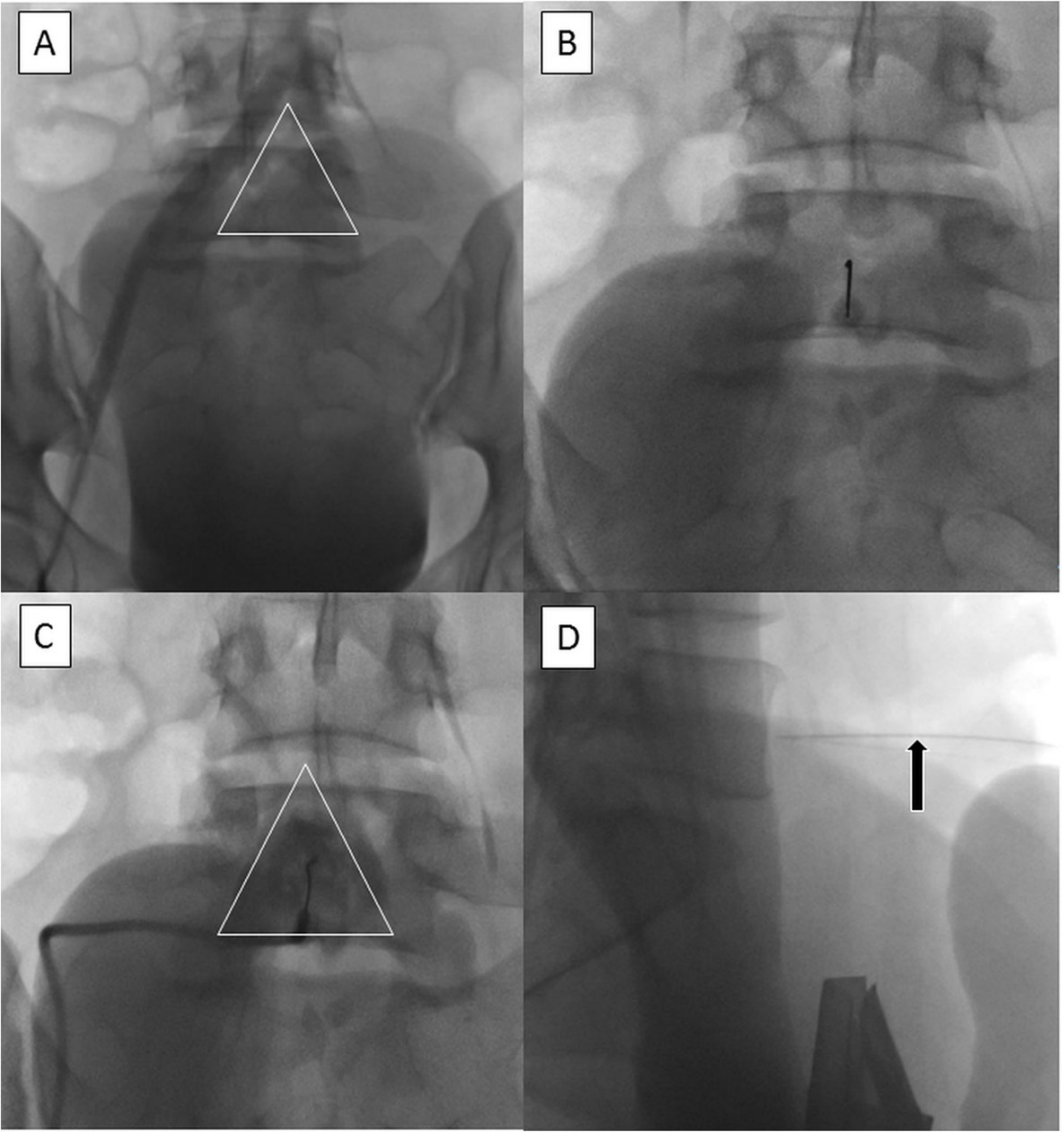
SNHB after UFE **a** Abdominal aortogram (bone window) in a caudal projection delineates the aortic bifurcation and iliac vessels as well as L5 vertebral body. An ideal location is a triangular area below the bifurcation (white triangle) **b** A 21 G needle is advanced anteriorly till bony resistance is felt when it contacts the anterior L5 vertebral margin. **c** Contrast injection shows the characteristic triangular blob of contrast (white triangle) with no vascular opacification. **d** The imaging intensifier is them moved to a lateral position to confirm position of tip off needle (black arrow) abutting the anterior margin of L5 vertebral body

The area of the abdomen below the umbilicus is prepped and draped in sterile fashion. Local anesthesia is administered to the skin. The entry site of the needle is visualized using a radiopaque object (like a hemostat) on the skin, typically 5–15 cm below the umbilicus. A 21/22-gauge Chiba (Cook medical Inc.) needle is advanced to the anterior portion of the 5th vertebral body under ongoing fluoroscopic guidance (Fig. 3). Once bony resistance is reached, using a connecting tube 2–5 ml slightly dilute contrast is gently injected which typical reveals a characteristic triangular blob of contrast with no vascular opacification. The Flat-panel is then positioned in a lateral view and contrast injected. Again typically, a crescent contrast area directly in front of the vertebral body is seen.

#### Choice and technique of anesthetic injection

An ideal anesthetic agent for SHNB after UFE is one that is effective in relieving pain and cramping that typically worsen during the first 2 to 3 h, reaching a constant level for 8 to 12 h and then subsides (Spencer et al. 2013). Vascular injection of a local anesthetic can result in central nervous system and cardiovascular toxicity, especially relevant in SHNB which is performed from an anterior approach.

Bupivacaine (trade name Marcaine) is a long-acting local anesthetic agent that has been used for regional anesthesia including SHNB (Rasuli et al. 2004; Spencer et al. 2013; Park et al. 2020). Levobupivacaine (Chirocaine, AbbVie Ltd) and ropivacaine (Naropin, AstraZeneca) are two S-enantiomers of bupivacaine which were developed more recently to reduce cardiac and central nervous system toxicity. They have similar onset, duration and potency of sensory block to bupivacaine but with a safer pharmacological profile. They are less lipophilic and are therefore less likely to penetrate large myelinated motor fibers, which decreases the potential for central nervous system toxicity and cardiac toxicity (Kuthiala and Chaudhary 2011). Typically, onset of sensory block is within the first hour with duration of up to 12 h of injection (Park et al. 2020).

After confirmation of a good position and extravascular location of the tip of the needle, injection is initiated. Before injection the needle is aspirated to confirm there is no blood. A preliminary test dose of about 3 cc of 0.5% Ropivacaine is then injected. If there is no change in heart rate or neurological status, rest of the 17 cc of the total 20 cc (total of 60 mg of Ropivacaine) is injected slowly with intermittent aspiration. During the entire injection, a slight forward tension is kept on the needle in order to avoid retraction into other structures including veins.

Reported series of SHNB have used various local anesthetic agents in different strengths and volumes. Rasuli et al. used 20 mL 0.25% bupivacaine (Rasuli et al. 2004), Spencer et al. 15-20 mL 0.5% bupivacaine (Spencer et al. 2013), Binkert et al. 10 mL 0.75% ropivacaine (Binkert et al. 2015) and Park et al. 20 mL 0.25% bupivacaine (Park et al. 2020).

### Technical challenges and troubleshooting

Although technical success of SHNB was high (87%) in our study, technical failures can occur (Pereira et al. n.d.).

**Fig. 4:**
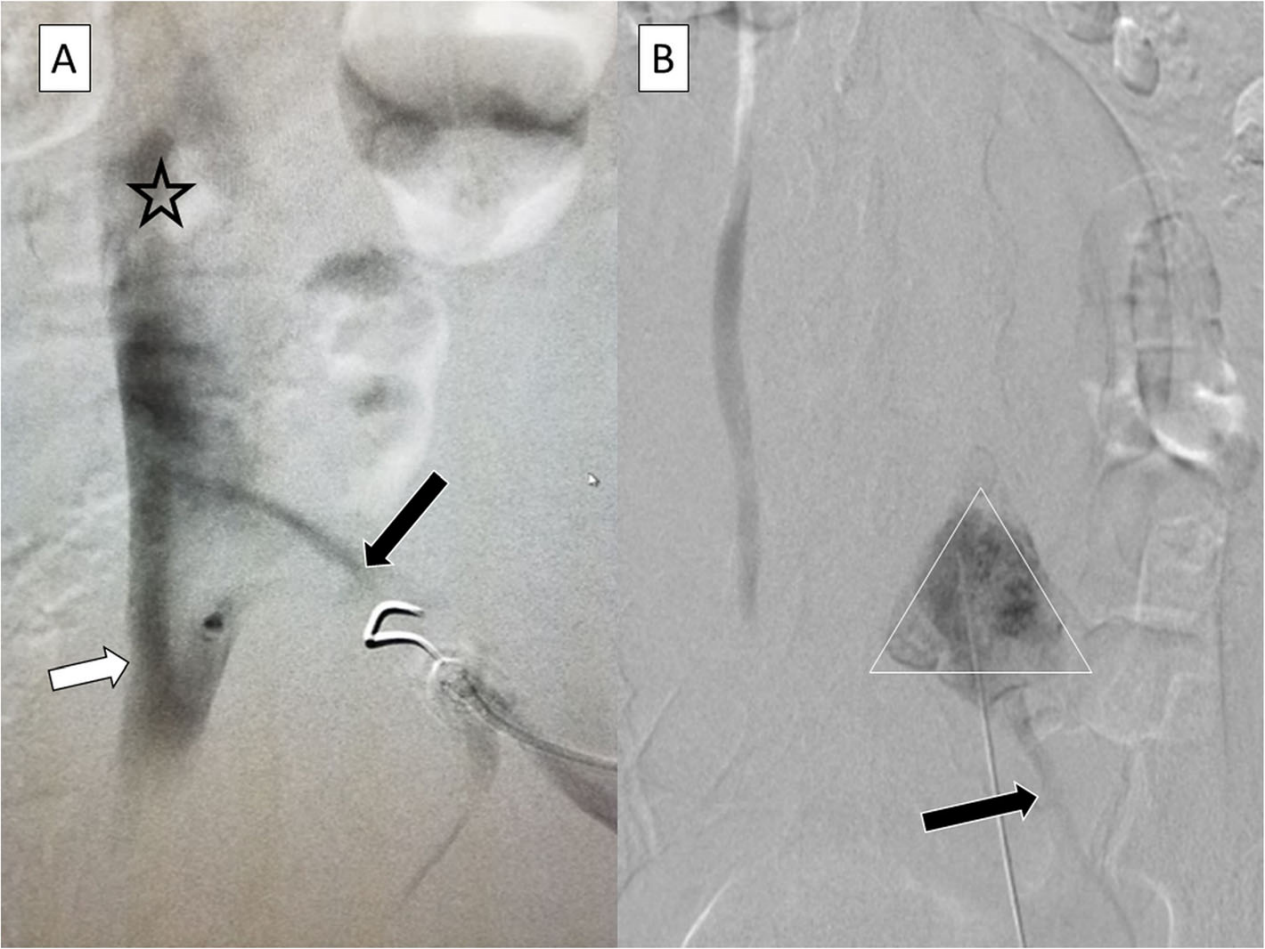
While arterial opacification can be avoided by performing an aortogram and defining arterial vascular anatomy, it is difficult to define venous outlines and inadvertent venous opacification can occur. **a** Shows needle in the left common iliac vein (black arrow) with opacification of the IVC (black asterisk) and even contralateral common iliac vein (white arrow). **b** The a characteristic triangular blob of contrast for SHNB is seen (white triangle). However the external iliac vein also opacified (black arrow). Needle was repositioned and SHNB performed

- Traversing the large fibroid uterus: UFE is often performed for large fibroid uteri, often in the percutaneous needle path to the L5 vertebra. A 20 G needle (Chiba -Cook medical Inc.) and sometimes an 18 G is preferred over a 21/22 G for better steerability and penetration, 15–20 cm length is often the best choice. In our experience case cohort, we traversed the uterus in 65% times (*n* = 27/41). We had to reposition the needle 36% cases (*n* = 11/30).
- Vascular opacification: Although arterial opacification is not commonly seen, venous opacification, in our experience was observed in 17% cases (*n* = 7/41). This is not surprising as anatomically the IVC confluence/ interiliac angle resides at the L5 level. Potential reasons that venous opacification is not observed more often is that the needle possibly traverses at a relatively ‘flattened’ interiliac angle, traversing venous structures are ‘collapsed’ in a state of relative dehydration due to fasting, and venous compression due to fibroids. Of the 7 incidences of venous opacification, the left common iliac vein was opacified in 5 cases and the inferior IVC/ iliac confluence in 2. (Fig. 4) If vascular opacification is seen it is important to reposition the needle prior to local anesthetic injection
- Local Anesthetic Systemic Toxicity (LAST): LAST can occur in the setting of an unintentional intravascular injection.. Symptoms reflect CNS and cardiac toxicity and include perioral tingling, slurred speech, convulsions, hypertension and tachyarrhythmias. Cardiovascular collapse, respiratory depression and coma can result. Following identification of LAST, airway support, seizure suppression, Advanced Cardiac Life Support protocol and lipid emulsion (20%) therapy should be initiated (Rubin et al. 2018). The hypothesized mechanism of lipid emulsion is that the emulsion binds to the lipophilic local anesthetic, preventing it from binding to cardiac or nerve receptors.

## Conclusion

(SHNB has been shown to be effective pain suppressant after UFE, reducing the need for opiates and allowing same-day discharge after UFE. It can be a useful tool in the Interventional armamentarium to make UFE a better experience for patients with fibroids. Performing SHNB appear to be can be performed with technical ease for an interventional radiologist.

## Data Availability

Not applicable.

## Abbreviations

UFE: Uterine Fibroid Embolization
SHNB: Superior Hypogastric nerve Block
IVC: Inferior Vena Cava
LAST: Lidocaine Associated Systemic Toxicity
SHP: Superior hypogastric plexus
